# Hard shell, soft blue-green core: Ecology, processes, and modern applications of calcification in terrestrial cyanobacteria

**DOI:** 10.1016/j.isci.2024.111280

**Published:** 2024-10-28

**Authors:** Patrick Jung, Laura Briegel-Williams, Stefan Dultz, Carina Neff, Gunnar Heibrock, Curtis Monger, Nicole Pietrasiak, Lena Keller, Julia Hale, Jan Friedek, Timo Schmidt, Georg Guggenberger, Michael Lakatos

**Affiliations:** 1 University of Applied Sciences Kaiserslautern, Integrative Biotechnology, Carl-Schurz-Str. 10-16, 66953 Pirmasens, Germany; 2 Leibniz Universität Hannover, Institute of Earth System Sciences, Section Soil Science, Herrenhäuser Str. 2, 30419 Hannover, Germany; 3 University of Applied Sciences Kaiserslautern, Building and Design, Schoenstr. 6, 67659 Kaiserslautern, Germany; 4 New Mexico State University, Plant and Environmental Science, Las Cruces, NM, USA; 5 University of Nevada, Life Science Department, Las Vegas, NV, USA; 6 Weincampus Neustadt, University of Applied Sciences Kaiserslautern, Dienstleistungszentrum Ländlicher Raum Rheinpfalz, Breitenweg 71, 67435 Neustadt a.d. Weinstraße, Germany; 7 University of Applied Sciences Augsburg, Architecture, An der Hochschule 1, 86161 Augsburg, Germany

**Keywords:** Geochemistry, Biogeochemistry, Ecology, Microbiology

## Abstract

Cyanobacteria are the oldest photoautotrophic lineage that release oxygen during photosynthesis, an ability that possibly evolved as far as 3.5 billion years ago and changed the Earth’s environment—both in water and on land. Linked to the mechanism of carbon accumulation by cyanobacteria during photosynthesis are their calcifying properties, a process of biologically mediated mineralization of CO_2_ by precipitation with calcium to CaCO_3_. In recent decades, scientific research has mainly focused on calcifying cyanobacteria from aquatic habitats, while their terrestrial relatives have been neglected. This review not only presents the ecology of terrestrial calcifying cyanobacteria in caves and biocrusts but also discusses recent biotechnological applications, such as the production of living building materials through microbial-induced carbonate precipitation for structural engineering, which has the potential to open a new and efficient pathway for mitigating climate change, e.g., as carbon capture and storage technology.

## Graphical abstract

**Figure:**
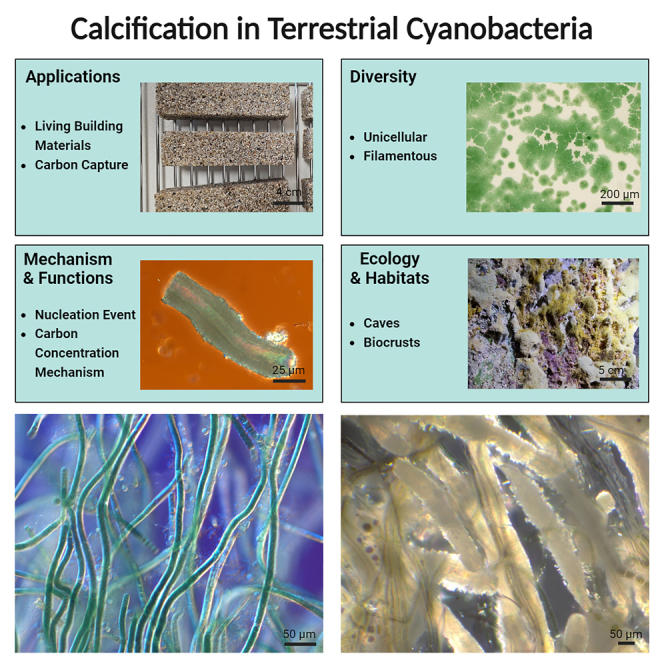

## Introduction

**Figure 1:**
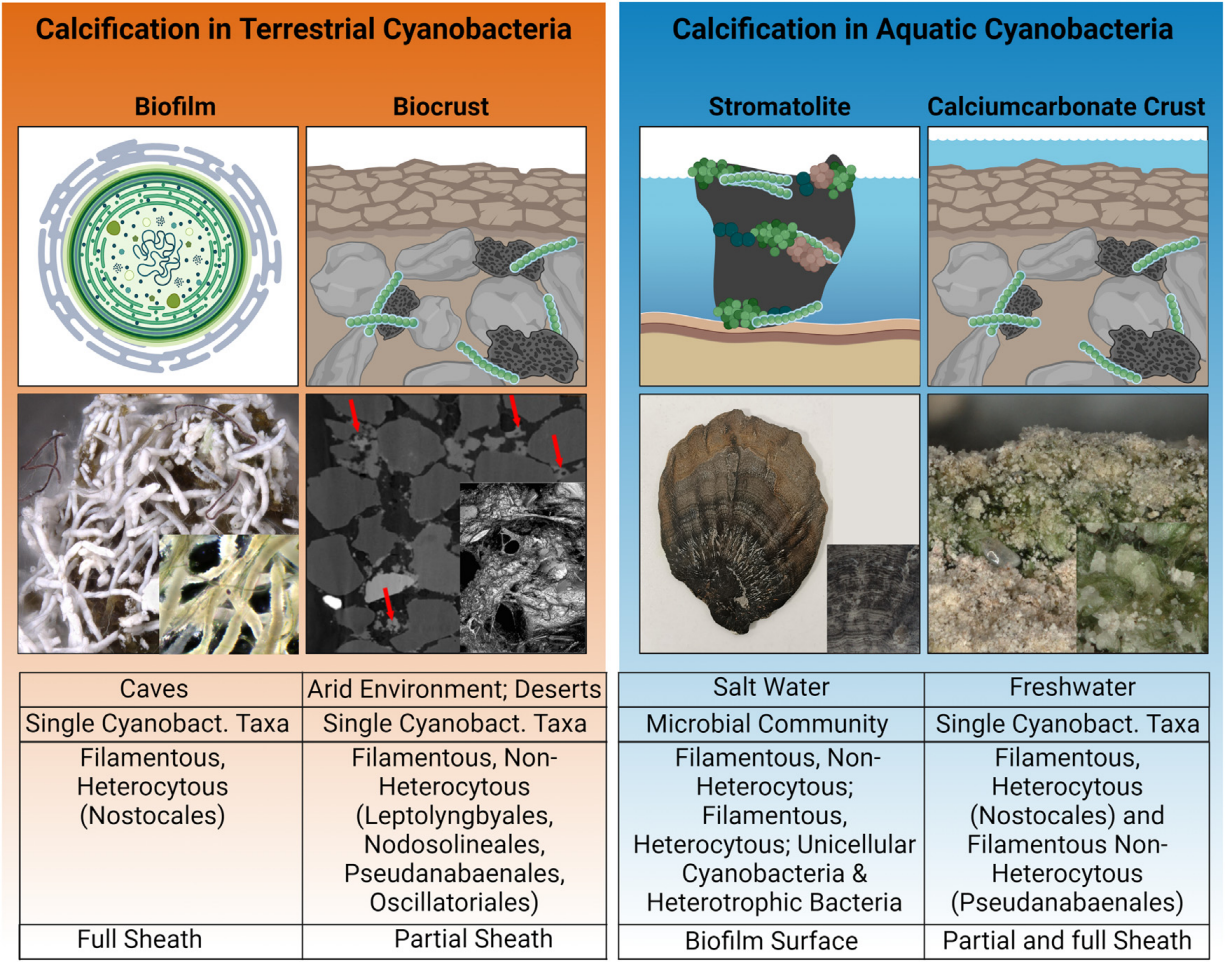
Comparison of cyanobacterial calcification between aquatic and terrestrial habitats Top column gives information about the habitat, second row about the main responsible calcifier, third row gives the main taxa involved in the calcification process, and bottom row gives the mode of calcification.

Microbial calcification, often referred to as microbial induced carbonate precipitation (MICP), the process of biologically mediated mineralization of CO_2_ resulting in the precipitation of calcium carbonate (CaCO_3_) based on a nucleation event, is a widespread phenomenon in aquatic and terrestrial ecosystems (Figure 1). It plays a crucial role in global biogeochemical carbonate and carbon cycles.^1^^,^^2^ It is estimated that the current production of CaCO_3_ in the worlds oceans is about 5–5.6 billion tons per year,^3^ while data for terrestrial systems are lacking. Two types of calcification can be distinguished: biomineralization, which is biologically controlled (e.g., organisms actively regulating the deposition of minerals), and organomineralization, which is biologically induced or influenced (e.g., organisms influencing the surrounding environment to precipitate minerals).^4^ The majority of the Earth’s carbonates are biogenic and result from microbially mediated CO_2_ precipitation.^5^ Prior to the Cambrian explosion of life, more than 500 million years ago, stromatolites, which are laminated carbonate structures, were prevalent in shallow marine seas.^6^ These structures can still be found today, for example in Shark Bay (Australia), Alchichica (Mexico), the Salar de Atacama (Chile), Bogoria (Kenya), and many other sites.^7^^,^^8^

Calcification is best known for its role in deep-sea carbon storage due to planktonic calcifying (micro)organisms, including various microalgae that sink to the ocean floor after dieback.^9^ Many studies focus on the marine coccolithophore *Emiliania huxleyi* as a model organism for unicellular calcification and its role in oceanic carbon capture during climate change and ocean warming.^10^^,^^11^

Microbial biomineralization of CaCO_3_ in aquatic environments has been recognized for a long time.^12^ In contrast, pedogenic CaCO_3_ has traditionally been viewed as an inorganic physicochemical phenomenon. Only in the 1970s, with the advent of scanning electron microscopy, did the potential role of microorganisms become progressively recognized.^13^^,^^14^ As a result, calcification was observed not only for soil bacteria^15^ and fungi^16^ but also for aerophytic cyanobacteria. Although the principle metabolic mechanisms mediating the cyanobacterial calcification process of aquatic and terrestrial cyanobacteria might be comparable, it is unlikely that terrestrial species have the same ecological advantages concerning a carbonated, solid sheath. This is likely explained by differences in environmental interaction, metabolism, and excess production of extracellular polymeric substances (EPS; with high abundance of predominantly negatively charged biomolecules, including amino acids, e.g., aspartate, glutamate, and saccharides, e.g., glucuronic acid, mannuronic acid, and Ca^2+^ and Mg^2+^ ions for charge neutralization and bridging between the organic molecules) of terrestrial species. Only speculations exist regarding the ecological advantages of such solid sheaths, and moreover, it is unclear why some cyanobacteria produce such CaCO_3_ sheaths while others do not or how these sheaths have provided competitive advantages during the long evolutionary history of the phylum.

Cyanobacteria are the oldest known photoautotrophic organisms that release oxygen during photosynthesis. This ability possibly evolved about 3.5 billion years ago and resulted in the “Great Oxidation Event” (GOE) 2.4 billion years ago,^17^ which forever altered the Earth’s environment.^18^^,^^19^^,^^20^ Since their origin, cyanobacteria have constantly evolved and adapted to diverse habitats on Earth. They can be found in saline ocean environments, oligotrophic to eutrophic freshwater bodies, and even the driest, hottest, and coldest deserts.^21^ Additionally, they have formed symbiotic relationships with fungi, diatoms, bryophytes, ferns, and vascular plants.

Terrestrial calcifying cyanobacteria have been found in various habitats, including caves in Italy and France,^22^^,^^23^ rock walls in Florida,^24^ and as epiphytes in Papua New Guinea.^25^ These calcifiers are typically found near potential sources of Ca^2+^, such as limestone, karst, or even bone material (see Table 1).

**Table 1:** List of aerophytic cyanobacteria reported to mediate calcification

Species	Location	Source
*Geitleria calcarea*	Limestone caves, Jerusalem Limestone caves, Poiters, France Caves, Atlantic Pyrénées, France	Friedmann^26^ Bourrelly and Dupuy^22^
*Geitleria floridana*	Cave walls, Florida, USA	Friedmann^26^
*Geitleria appalachiana*	Limestone walls, USA	Kilgore and Johansen^24^
*Scytonema julianum*	Cave wall, Necropolis tomb, Greece Cave walls, Spain	Ariño et al.^23^
*Scytonema* sp.	Aerophytic habitats, Papua New-Guinea	Hoffmann^25^
*Loriella osteophila*	Human bones, New Guinea Limestone, Papua New-Guinea	Borzi^27^ Hoffmann^28^
*Pleurocapsa* spp.	Stromatolites, Borrego Desert, USA	Krumbein and Giele^29^
*Loriellopsis cavernicola*	Caves, Spain/Greece	Lamprinou et al.^30^
*Iphinoe spelaeobios*	Caves, Spain/Greece	Lamprinou et al.^30^
*Iphianassa zackieohae*	Cave, Greece	Panou and Gkelis^31^

The cyanobacteria-facilitated calcification processes may also hold significant importance within biocrusts, where both soil inorganic and organic components are linked together by (1) the filamentous structure of cyanobacteria and other microorganisms, (2) their EPS with gluing properties in biofilms, and (3) their capacity for calcification, potentially leading to the cementation of soil structures either directly or by solution-precipitation mechanisms. This phenomenon enables cyanobacteria-dominated biocrusts to mitigate wind or water erosion, a critical ecosystem service, particularly in semi-arid and arid environments where they are prevalent.^32^

The evolution of oxygenic photosynthesis in cyanobacteria, resulting in a decrease in atmospheric CO_2_, also led to the development of an efficient photosynthetic CO_2_ concentrating mechanism (CCM),^33^ which appears to be linked to cyanobacteria’s ability for calcification. Extensive research conducted over recent decades on the calcification process mediated by cyanobacteria suggests a complex interplay between cyanobacterial metabolism and their immediate environment (reviewed by Kamennaya et al.^34^). Nonetheless, questions persist regarding whether the mucilaginous EPS serves as the site for nucleation and CaCO_3_ crystallization initiation, as well as how the cell wall, EPS, and solidified sheath functionally interact within this process in contrast to intracellular CaCO_3_ biomineralization.^35^

One of the central inquiries regarding terrestrial cyanobacterial biomineralization pertains to the overarching purpose of this ability, as only speculative hypotheses exist regarding the ecological benefits of “self-entombment” with CaCO_3_.^34^^,^^36^

Despite the uncertainties surrounding the precise cellular mechanisms involved, cyanobacterial calcification abilities are increasingly utilized in the field of applied sciences for biotechnological purposes. Various bioreactor techniques have been developed, enabling the commercial large-scale cultivation of cyanobacterial strains and subsequent directed calcification, yielding living building materials (LBMs) like concrete with a negative CO_2_ balance.^37^^,^^38^ Questions within this relatively new research area arise, for example, regarding the factors that influence the long-term stability of such LBMs or the commercial value. This review article aims to (1) explore the ecology of calcification mediated by terrestrial cyanobacteria, focusing on environments such as caves and biocrusts, (2) evaluate the conflicting state of knowledge regarding the metabolic biochemical processes involved, and (3) present biotechnological applications in structural engineering and the mitigation of erosion and desertification.

## A different world: Ecology of terrestrial cyanobacterial calcification

Calcification facilitated by cyanobacteria manifests itself differently in aquatic and terrestrial environments. In aquatic environments, cyanobacteria-dominated microbial mats, including various microorganism groups,^39^^,^^40^ can form rounded, stratified structures composed of cells encased in CaCO_3_, or living biofilms known as stromatolites or microbialites, which can span several meters in diameter.^41^ In contrast, the formation of CaCO_3_ sheath material in terrestrial environments is predominantly attributed to individual cave or biocrust-inhabiting filamentous, heterocytous, and often true-branching cyanobacterial taxa, rather than entire communities (see Table 1; Figure 1). In these environments, dense, typically white structures of fine crystals develop around the filaments, with varying thicknesses and crystal orientations.^23^^,^^25^^,^^30^^,^^42^ Because terrestrial habitats, such as caves or biocrusts, provide contrasting conditions compared with aquatic environments where stromatolites are predominantly formed (Table 2), the ecology of these cyanobacteria differs significantly from their aquatic relatives. Most importantly, terrestrial cyanobacteria often produce large amounts of EPS as a strategy that helps them to move, exchange metabolites, and protects them from desiccation and strong radiation (reviewed in Rossi and De Philippis^43^). Simultaneously, the EPS matrix provides an additional site for interactions within the cell’s direct environment and allows modifications such as providing a nucleation site for the formation of CaCO_3_ sheaths, which is one of the proposed mechanisms during cyanobacterial calcification.

**Table 2:** Minerals involved in the calcification process in their natural aquatic and terrestrial environment and under laboratory conditions

Cyanobacteria	Location	Mineral	Reference
*Dichothrix* sp.	Marine stromatolites (Bahamas)	Aragonite/Calcite	Planavsky et al.^9^
*Gloeocapsa* sp.	Alkaline lake (Turkey)	Hydro magnesite	Braithwaite and Zedef^44^ Braithwaite and Velsel^45^
*Homoeothrix crustacea*	Calcareous streams (England)	Calcite	Pentecost and Bauld^46^
*Lyngbya* sp.	Alkaline wetland (Canada)	Aragonite/Dypingite	Power et al.^47^
*Phormidium* sp. and *Oscillatoria* sp.	Fresh water,	CaCO_3_	Dupraz et al.^4^
*Phormidium* sp.	Lakes (USA)	CaCO_3_	Arp et al.^48^
*Phormidium* sp.	Lakes (China)	Aragonite	Arp et al.^49^
*Phormidium incrustatum*	Creeks (Germany)	CaCO_3_	Shiraishi et al.^50^
*Plectonema gloeophilum*	Freshwater pool (Seychelles)	CaCO_3_	Riding,^51^
*Pleurocapsales*	Alkaline brackish caldera lake (Mexico)	Aragonite/Hydromagnesite	Kaźmierczak et al.^46^
*Rivularia hematites*	Calcareous water streams (England)	Calcite	Pentecost and Talling^52^
*Scytonema* sp*./Schizothrix* sp.	Freshwater prairies (USA)	CaCO_3_	Thiel et al.^53^
*Geitleria appalachiana*	Limestone walls (USA)	CaCO_3_	Kilgore and Johansen^24^
*Geitleria calcarea*	Terrestrial cave walls (France)	CaCO_3_	Couté^54^
*Geitleria floridana*	Terrestrial cave walls (USA)	–	Friedmann^26^
*Loriella osteophila*	Limestone walls (Papua New Guinea)	–	Hoffmann^25^
*Loriella osteophila*	Cave walls (Spain)	CaCO_3_	Roldán^55^
*Lyngbya incrustatum*	Travertine (England)	CaCO_3_	Pentecost^56^
*Scytonema mirabile*	Lacustrine sediment (French Polynesia)	Mg-calcite	Défarge et al.^41^
*Scytonema* sp.	Tree bark (Papua New Guinea) Limestone (Papua New Guinea) Soil samples (Papua New Guinea)	Acicular CaCO_3_ Dendritic CaCO_3_ Triradiate CaCO_3_	Hoffmann^25^
*Scytonema julianum*	Terrestrial cave walls (Italy)	Calcite	Ariño et al.^23^
*Scytonema julianum*	Terrestrial stone wall (Cayman Island)	Amorphous CaCO_3_ Acicular crystals	Jones and Peng^42^
*Entophysalis major*	Lab	Calcite	Pentecost and Bauld^57^
*Leptolyngbya* sp.	Lab	Calcite	Shiraishi et al.^58^
*Lyngbya aestuarii*	Lab	Calcite	Pentecost and Bauld^57^
*Phormidium* sp.	Lab	Calcite/Amorphous CaCO_3_	Shiraishi et al.^58^
*Pseudanabaena minuta*	Lab	Calcite	Pentecost and Bauld^57^
*Scytonema* sp.	Lab	Amorphous CaCO_3_	Shiraishi et al.^58^
*Scytonema myochorus*	Lab	Calcite	Pentecost and Bauld^57^
*Spirulina* sp.	Lab	Calcite/Amorphous CaCO_3_	Shiraishi et al.^58^

In general, calcified structures such as tubes formed by microscopic life forms remain during the fossilization process, and many of these findings have been assigned to cyanobacteria (Table 3; reviewed in Riding^59^). Calcified cyanobacterial fossils have long been observed in limestone, although they have often been confused with other organisms.^60^ Microfossils may provide direct evidence for cyanobacteria, but their identification is often ambiguous. The identity of only a few cyanobacterial microfossils has been confirmed (albeit not forming calcified sheaths) such as *Eoentophysalis*, *Eohyella*, and *Polybessurus*.^61^ *Eoentophysalis belcherensis*, for example, is the oldest microfossil interpreted with certainty as a cyanobacterium dated to 1.89–1.84 Ga from silicified stromatolites of the Belcher Supergroup, Hudson Bay, Canada.^62^ Such findings can help with phylogenetic clock calibrations, dating of global events, or the evolution of early photosynthesis (reviewed in Demoulin et al.^61^), thus they are a valuable resource.

**Table 3:** List of potential fossil cyanobacteria reported to mediate calcification

Species	Location	Growth form	Dating
*Girvanella*	Lunnan, Tarim, China	Tubular	Early mid-Ordovician
*Batinivia*	England	Cable-like strands	Lower Carboniferous
*Botominella, Cladogirvanella, Razumovskia, Subtifloria*	–	Cable-like strands	–
*Obruchevella*	–	Coiled, cable-like strands	–
*Ortonella*	England	Spherical stromatolite nodules, Erect, branched	Lower Carboniferous
*Angusticellularia*	Sibiria	Dendritic shrub-like mass of dense micrite	Cambrian
*Epiphytonoides, Gordonophyton, Tubomorphophyton*	–	Narrow branched micritic filaments, not tubiform	–
*Renalcis*	Sibiria	Densely micritic branched filaments, hollow chambers	Cambrian
*Izhella, Shuguria*	–	Densely micritic branched filaments, several hollow chambers	–
*Chabakovia*	–	Densely micritic branched filaments, several hollow chambers, thin walls	–
*Gemma, Tarthina*	–	Densely micritic branched filaments, several hollow chambers, thick walls	–

### The cave environment: Solid sheath formation by Geitleria and Scytonema

**Figure 2:**
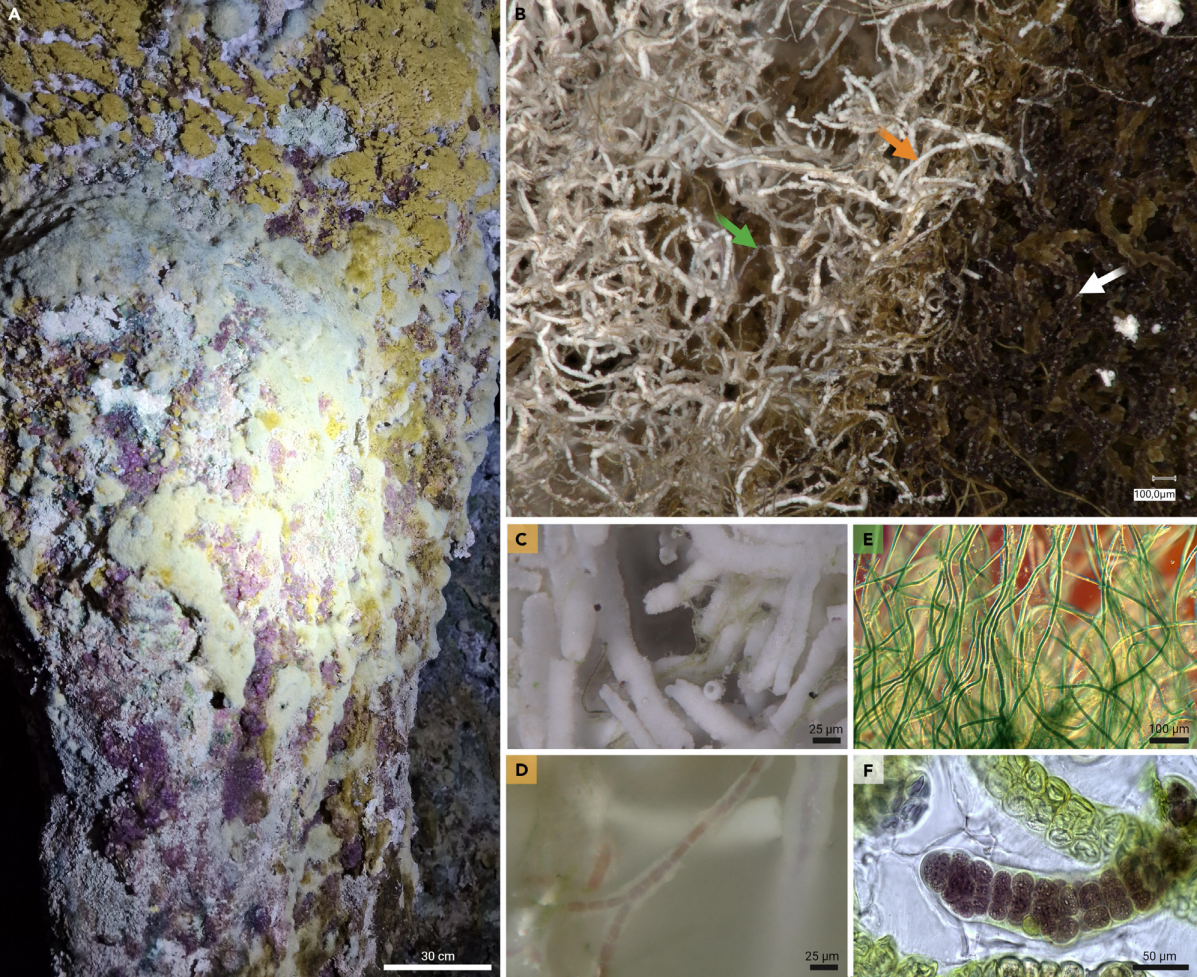
The cyanobacterial cave environment (A) Cyanobacterial biofilm with various taxa forming different growth structures and colorations in patches from a cave wall in Northern Spain. All pictures were taken by the authors. (B) Close-up of a layered patch showing a top layer of whitish calcified sheath of the cyanobacterium *Geitleria* sp. (orange arrow) intermixed with filaments of *Scytonema* sp. (green arrow) as well as a bottom layer of non-calcified thick filaments of *Stigonema* sp. (white arrow). (C) Close-up of tubular CaCO_3_ sheaths formed by *Geitleria* sp. (D) Microscopic image showing the tubular CaCO_3_ sheaths of *Geitleria* sp. as well as the true-branched, red filament inside the solid sheath. (E) Microscopic image of non-calcified filaments of *Scytonema* sp. (F) Microscopic image of non-calcified filament of *Stigonema* sp.

Special attention should be directed toward terrestrial cyanobacteria inhabiting cave systems, as biomineralization is often observed on a larger scale in these environments (Figure 2). Biofilms are commonly observed on limestone walls, where marked bio-weathering effects are evident.^63^^,^^64^ This suggests that cells may actively utilize minerals from their environment for element mining and may even incorporate them in biomineralization processes, particularly for calcified sheaths.^65^^,^^66^ Besides a significant Ca^2+^ source, also high (slightly alkaline) pH is needed for calcification. Since rates of Ca^2+^ release and proton buffering by silicates are typically low, calcified sheath formation in biofilms is typically limited to carbonate rocks.

These adapted cyanobacteria can thrive in low-light conditions, with some exhibiting robust biomineralization capabilities.^23^^,^^67^ Cave systems can be colonized by naturally occurring phototrophic microbial communities.^55^ Moreover, a specific and distinct "Lampenflora" can develop due to artificial lighting in tourist caves.^68^ According to Roldán and Hernández-Mariné,^69^ such phototrophic biofilms in caves can be categorized into different functional types based on their substrate, species dominance, and depth distribution within the biofilms: (1) encompasses several taxa and generally exhibits stratification, with a continuous upper layer of chlorophytes and diatoms and a discontinuous bottom layer of unicellular and filamentous cyanobacteria. (2) Formed by very few photosynthetic organisms but remains stratified with unicellular cyanobacteria in the bottom and chlorophytes on top. (3) Formed by one or very few chlorophytes with heterotrophic bacteria, without stratification. (4) Formed by mosses or moss protonemata plus other photosynthetic organisms such as diatoms. (5) Formed by mainly lichens. (6) Structured by mainly calcified cyanobacteria in addition to other filamentous and unicellular cyanobacteria. The latter is depicted in Figure 2B where a top layer is formed by the true-branching, heterocytous *Geitleria* sp. forming large, whitish tubular CaCO_3_ sheaths (Figures 2C and 2D), intermingled with less calcifying filaments of the false-branching *Scytonema* sp. (Figure 2E). Underneath, a bottom layer forms by the true-branching, multiseriate *Stigonema* sp. without any solidified EPS sheath (Figure 2F).

**Figure 3:**
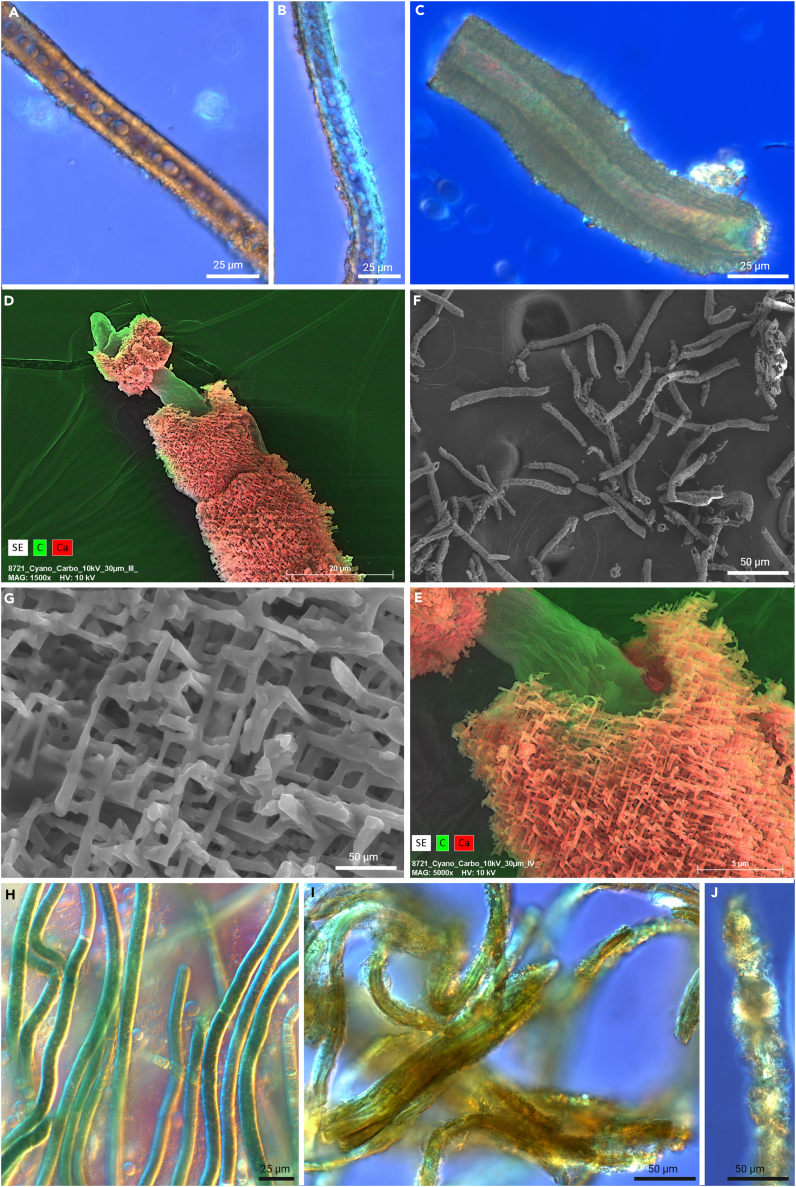
Calcifying cyanobacteria *Geitleria* sp. and *Scytonema* sp. from cave environments (A, B, and C) Microscopic images showing filaments of *Geitleria* sp. without a calcified sheath in (A), weak calcified sheath in (B), and a fully developed calcified sheath without a living filament in the tubular structure in (C). (D and E) SEM-EDX mapping showing details of the calcium-enriched sheath material of *Geitleria* sp. (F and G) SEM images of *Geitleria* sp. giving details of the mineral structure of the solidified sheath of *Geitleria* sp. (H, I, and J) Microscopic images showing filaments of *Scytonema* sp. without a calcified sheath in (H), scytonemin (brown) and calcium-enriched sheaths in (I) as well as a detail of a single filament with needle-like crystals alongside the sheath material in (J).

In general, calcifying species of *Scytonema*^25^^,^^42^ and *Geitleria*^24^^,^^55^ have been identified in terrestrial habitats worldwide. *Scytonema* species are primarily found at cave entrances with relatively high light exposure, whereas *Geitleria* spp. prefers habitats deeper within cave systems.^26^^,^^55^ Both can be distinguished by DNA sequencing techniques,^42^ but this is often a difficult process since the CaCO_3_ sheath material is mostly colonized by additional cyanobacteria, which results in mixed sequences during the sequencing process or inhibits molecular work due to interfering substances. Micro-manipulative methods such as direct PCR approaches^70^ have helped to overcome some of these issues and recently led to the molecular identification of *Geitleria* and *Scytonema* shown in Figures 2 and 3. In addition to modern phylogenetic differentiation approaches of calcifying cyanobacteria, delineation has also been undertaken based on morphological features, which are often well pronounced in such terrestrial cyanobacteria. For instance, *Geitleria* spp. typically exhibit thicker sheaths, ranging from 28 to 32 μm, whereas *Scytonema* species are associated with sheaths around 15 μm wide.^42^ In addition to cellular and sheath-related features, the crystal structure itself can vary significantly. Hoffmann,^25^ for example, described three different morphologies of the crystalline phases forming within (or in association with) cyanobacterial sheaths when studying terrestrial *Scytonema* spp.: triradiate flat crystals, triradiate elevated crystals, and acicular structures. Kilgore et al.^24^ gave additional information about the diversity of this process for *Geitleria appalachiana*. They described a lattice shaped, calcareous deposit of short, interwoven calcite needles, which is identical to the calcium-rich sheath of *Geitleria* sp. from caves of Spain in contrast to single-needle-like packages of long crystals in *Scytonema* sp. from the same habitat (Figure 3). Such crystals might be reminiscent of needle fiber calcite (NFC) or calcitic nanofibers where fungi seem to be involved in the process,^71^ but both types are much smaller (<500 nm) than the crystallized structures found associated with cyanobacteria. The formation of these cyanobacteria-associated crystals, at least in *Geitleria* sp., seems to be a graded process starting with juvenile filaments, free of mineral deposits followed by buildups of minerals until a thick sheath is created (Figures 2A–2C). *Scytonema* sp. seems to follow a different pattern, since a thick EPS sheath enriched with the UV-protecting, brownish pigment scytonemin is often formed followed by the formation of an incomplete carbonate sheath at a later stage (Figures 2H–2J). It was also reported that most crystal structures formed by e.g., *Scytonema julianum* can vary even between single aerial filaments of the same population or between different populations but at least the triradiate calcite crystals seemed to be species specific.^25^ Unfortunately, these observations have not been supported by molecular analyses. Additionally, there are no calcifying *Scytonema* strains currently in culture, nor has the isolation of *Geitleria appalachiana* been possible despite various attempts.^24^ This would allow further investigation of the process based on experiments targeting calcification (Table 2) involving the evaluation of the mineral composition.^72^

### The biocrust environment: Soil concatenated by cyanobacteria

In the uppermost millimeters of soils in habitats where abiotic conditions are not suitable for widespread vascular plant cover, a complex interplay of macroscopic lichens, bryophytes, microbial communities, and their byproducts leads to the formation of living soil aggregates known as biological soil crusts (biocrusts).^73^^,^^74^ Biocrusts are predominantly found in drylands and environments with various limiting environmental conditions, serving as hotspots of microbial biodiversity and activity. Hereby, the petrographic properties of the parent material of soil formation with, e.g., porosity, pore size distribution, mineral composition, Ca^2+^ release, pH-buffering, and color have a regulatory role for the habitat function.

**Figure 4:**
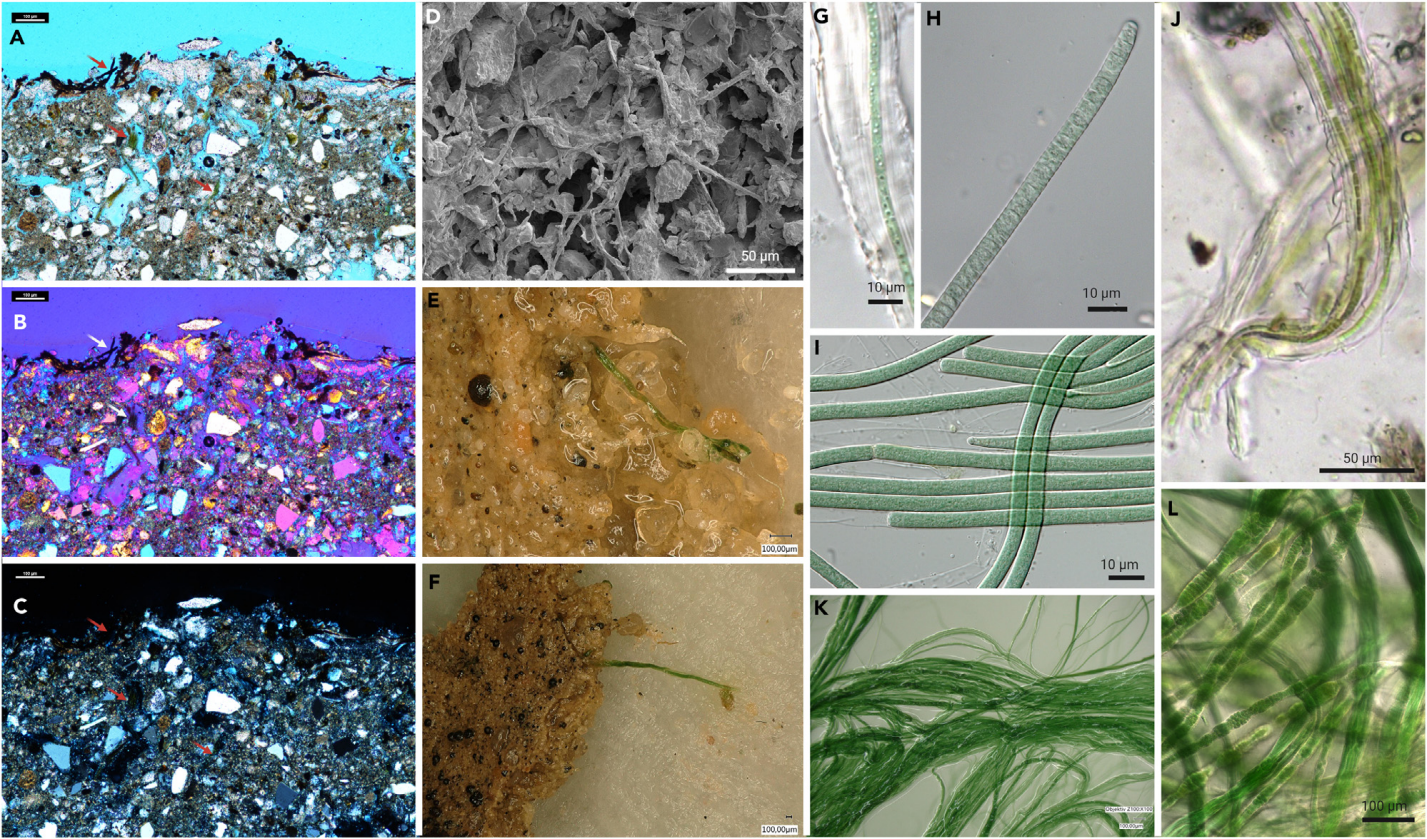
The filamentous cyanobacterium *Microcoleus* spp. associated with biocrusts across the globe (A–C) Microscopic thin section of biocrust visualized with petrographic filters highlighting pedogenic carbonates. Arrows indicate filamentous cyanobacteria. (D) SEM image of *Microcoleus* sp. from the European Alps concatenating mineral particles. (E and F) Filaments of *Microcoleus* sp. crawling out of wetted biocrust from the Sahara Desert, North Africa. (G–I) Different *Microcoleus* species from biocrusts of the Arctic Spitsbergen, Norway. (J) *Microcoleus* sp. associated with biocrusts in the sand dunes of the island Schiermonnikoog, Netherlands. (K and L) Bundle-forming *Microcoleus* sp. from biocrusts of the Atacama Desert, Chile.

EPS-producing cyanobacteria often initiate biocrust formation, with over 320 cyanobacterial species identified in biocrusts, representing at least 9 of the 19 recognized orders of cyanobacteria.^75^^,^^76^ Despite their unique biodiversity and essential functions, the process of calcification within biocrusts remains poorly understood. Currently, there aren't any cyanobacterial species assigned to biocrusts that have been found to form CaCO_3_ sheaths, but filamentous taxa such as *Microcoleus* spp. (Figure 4) are often the main stabilizer of such crusts where CaCO_3_ deposits are involved.^77^ They form rope-like, thick filaments with copious EPS sheaths that glue together minerals and act as microscopic wicks transporting water through pores and cracks where mineral crystallization is frequently observed.^78^ This genus is one of the most important taxa associated with biocrusts,^79^ representing at least 12 different lineages.^80^

**Figure 5:**
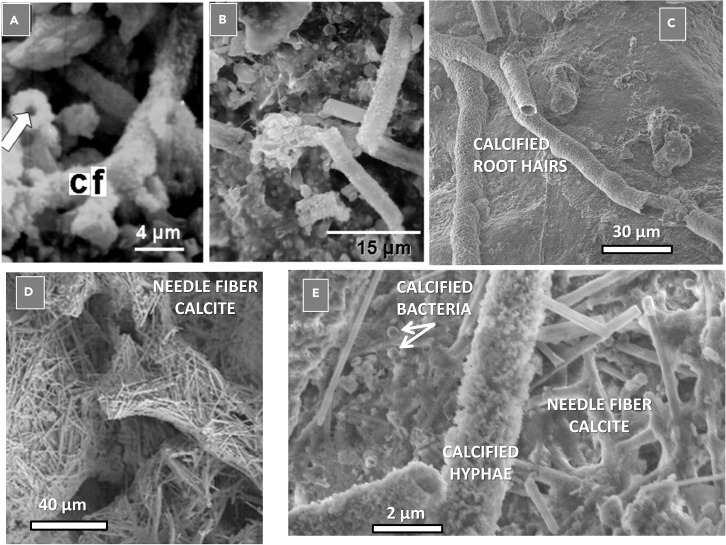
Complex calcification processes in the soil environment (A) Calcified fungal hyphae “CF” with arrow pointing to the characteristic hollow tubes. (B) Fragments of calcified fungal hyphae showing their typical diameter of approximately 4–5 μm. (C) Calcified root hairs distinguished by their larger diameter than fungal hyphae. (D) Acicular crystals of calcite diagnostic for needle fiber calcite formed in bodies of hyphae. (E) Image showing conglomeration of calcified hyphae, needle fiber calcite, and calcified spherical bacteria.

In addition to cyanobacteria, other microorganisms, such as fungi, which are frequently part of biocrusts, are known to possess calcification properties (Figure 5).

Calcification in biocrusts may be amplified compared to deeper soil layers due to higher microbial biomass and prolonged soil moisture availability in arid ecosystems, creating zones of heightened activity with increased respiration rates, and the presence of photoautotrophic microorganisms, including cyanobacteria. Calcification can occur via the general pathway through microbial respiration or the cyanobacteria-specific pathway involving CCMs. Additionally, a pathway specific to biocrusts, the calcium oxalate pathway, may also contribute to CaCO_3_ formation. Oxalic acid (H_2_C_2_O_4_), produced by various soil microbes within biocrust communities can react with free calcium ions to form calcium oxalate (CaC_2_O_4_). This compound can subsequently decompose via bacterial oxalotrophy, ultimately contributing to so-called caliche formation, a hardened natural cement of CaCO_3_ (sedimentary rock) with the ability to bind other materials such as gravel, sand, clay, and silt.^81^

Another factor contributing to the limited understanding of cyanobacterial calcification is the complexity of organic and inorganic carbon cycling pathways within the soil matrix of drylands. Light, CO_2_, Ca^2+^, and water availability in these soils differ significantly from aquatic habitats or other terrestrial environments. Light quantities and qualities within dryland soils can fluctuate rapidly and intricately highly depending on texture, with biocrusts primarily composed of sand exhibiting substantial light attenuation. While photosynthetic activity concentrates in the uppermost millimeters of soil, the presence of "light pockets" in vesicular pores (capillary wetting of the air-dry aggregate initiates the formation of vesicular micropores in the soil mass) may promote photosynthetic activity at deeper levels.^79^^,^^82^^,^^83^

Furthermore, belowground CO_2_ concentrations are significantly higher than in the aboveground atmosphere due to soil respiration, potentially impacting the photosynthetic efficiency of soil cyanobacteria and, consequently, calcification levels.^84^ However, the CO_2_ levels in dryland soils are lower than in other soils with a higher water content, as in the latter there is much higher biotic activity with higher CO_2_ production as a consequence of, e.g., respiration, and a higher share of pores are water saturated, thus reducing the convective and diffusive transport of CO_2_ out of the soil.

### Cyanobacterial calcification: A lifestyle?

**Figure 6:**
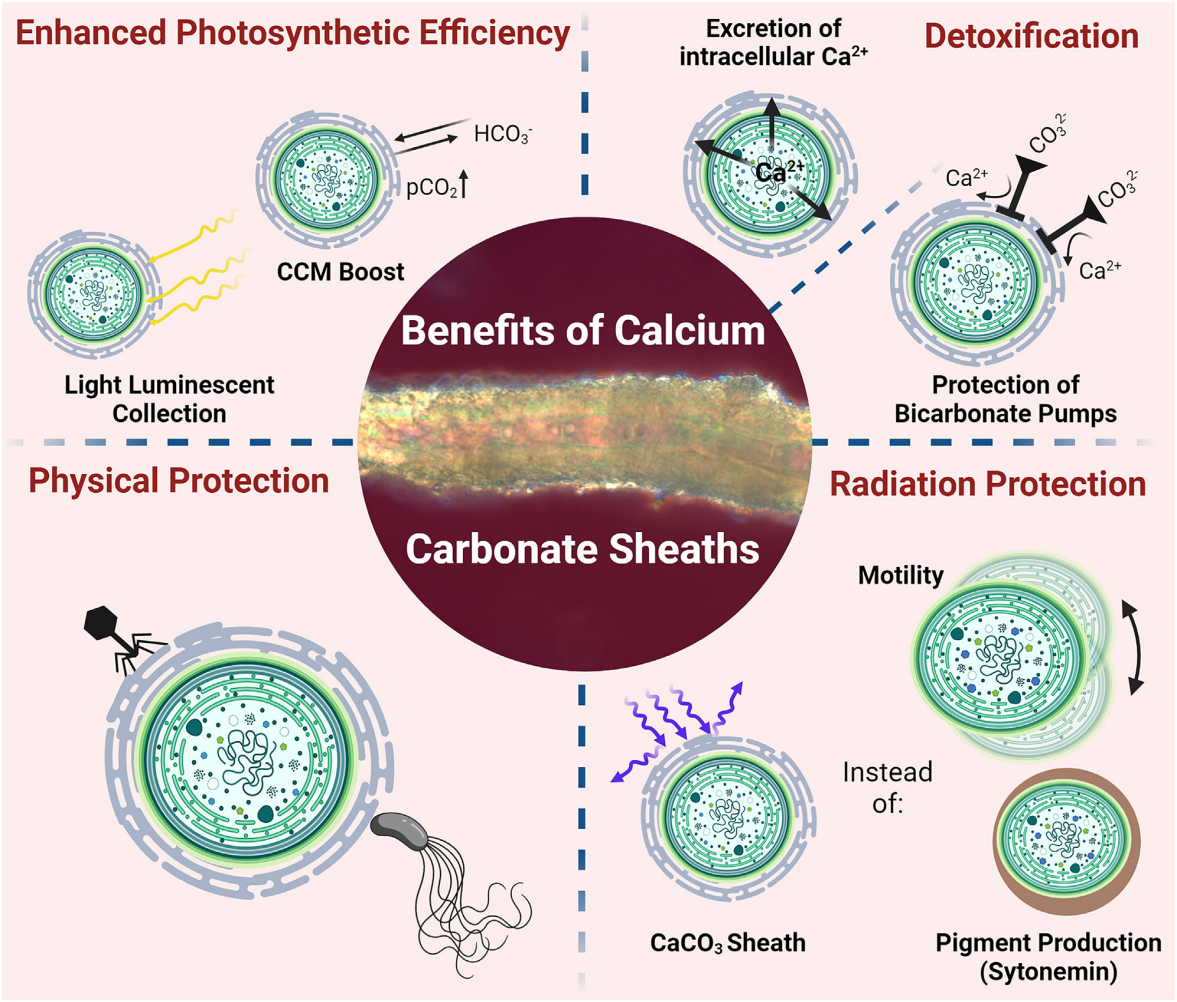
Overview of potential ecological advantages of cyanobacterial calcification Top left depicts possible advantages in photosynthetic efficiency by light trapping of the calcified sheath as well as a CCM boost. Top right shows the role of Ca-enriched sheaths as a consequence of a detoxification strategy by extracellular excretion and accretion of Ca^2+^ and additionally carbonate precipitation to protect bicarbonate transporters from carbonate ions. Bottom left indicates physical protection provided by the solid CaCO_3_ sheath against predators such as eukaryotes, viruses, or bacteria. Bottom right shows a potential radiation protection against UV and high light due to the calcified sheath as an alternative life strategy compared with motility or the production of pigments such as scytonemin.

There are numerous questions regarding biologically mediated calcification, including potential advantages cyanobacteria may derive from this process. In particular, if calcification is passive or actively mediated, why do certain taxa form calcified sheaths while others from the same habitat do not? To date, no clear evidence has demonstrated that the formation of calcified sheaths represents a cyanobacterial lifestyle.^85^ Instead, four main theories have been proposed (Figure 6): (1) physical protection, the CaCO_3_ sheaths protect against predators,^86^ (2) protection against high light intensities,^87^ (3) a light trapping mechanism to enhance photosynthesis,^88^ and (4) a detoxification process to eliminate Ca^2+^ from the cells^89^^,^^90^ and/or to protect the bicarbonate transporters from being blocked by carbonate ions.^91^

**Figure 7:**
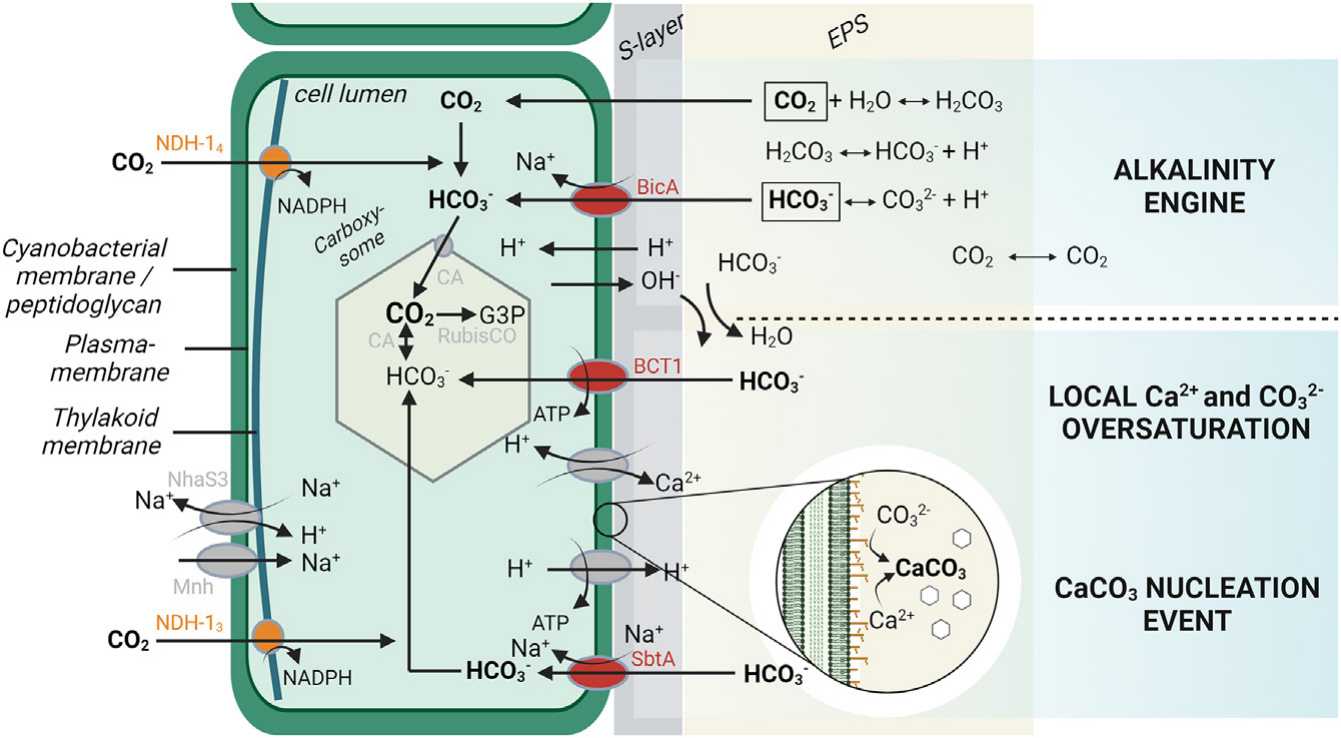
Molecular mechanisms involved in the cyanobacterial calcification process The BicA, SbtA, and BCT1 HCO_3_^−^ plasma membrane transporters (red) and the redox-powered NDH-1_3_ and NDH-1_4_ CO_2_ thylakoid membrane uptake systems (orange) are depicted. At low dissolved organic carbon levels, SbtA, BCT1, and NDH-1_3_ are activated, allowing for efficient transport of HCO_3_^−^ into the cytosol. Import of HCO_3_^−^ into the carboxysome and its subsequent dehydration back to CO_2_ by different carbonic anhydrase (CA) activities increases the local concentration of CO_2_ around RubisCO. Na^+^/H^+^ NADPH antiports and H^+^-ATPases are likely to be engaged to maintain intracellular Na^+^ and pH homeostasis. The Na^+^ gradients are kept by at least two Na^+^ export systems NhaS3 and the Mnh-complex (gray, left).

(1) Physical protection against predators (Figure 6, bottom left): one hypothesis suggests that CaCO_3_ sheaths may confer a selective advantage against predation or virus infections,^86^ which has recently been debated for both aquatic and terrestrial calcifying cyanobacteria.^92^^,^^93^ This protective effect has been observed for other calcifying organisms such as invertebrates and calcibacteria providing sponge symbionts with a sub-ectosomal cortex^94^^,^^95^ and additionally for dinoflagellates, which are coral symbionts.^96^ Laboratory experiments have indicated that cyanobacterial CaCO_3_ precipitation can be induced or influenced by podovirus-mediated cell lysis, suggesting an active defense strategy of the cells.^97^ However, this would imply that cyanobacteria not forming CaCO_3_ sheaths but co-occurring with those that do in the same environment would be particularly susceptible to virus infections and predation, which has so far not been shown, unless they employ different defense mechanisms. Moreover, the CaCO_3_ sheaths, composed of a large mineral matrix, significantly increase the surface area of cyanobacterial filaments and, together with the moist and sugar-rich EPS, may create a suitable habitat for viruses, bacteria, and ciliates that graze on different cyanobacterial sheath substances or the cells themselves.
(2) Physical protection against high light intensities (Figure 6, bottom right): UV protection is considered crucial for cyanobacteria, which have developed various protective mechanisms to prevent photodamage, such as the production of pigments like the yellow-brownish scytonemin (Figure 6), which is mostly produced by Nostocalean, heterocytous taxa such as *Nostoc*, *Scytonema*, or *Stigonema*.^98^^,^^99^ Alternatively, other cyanobacteria rarely invest in pigments, instead they are motile (Figure 6) such as the biocrust-assigned *Microcoleus*.^100^ This allows such taxa to adjust their position, e.g., within soil particles according to the light intensity.^101^ In addition, hypolithic (under translucent stones) or endolithic (in translucent stones) formations of cyanobacterial biofilms are lifestyles of certain cyanobacterial populations frequently observed in deserts where they are protected from strong light intensities or UV.^102^^,^^103^ An alternative theory proposes that extracellular calcium sheaths could serve as a protective shield against high irradiation and photoinhibition.^87^ The same has been proposed for the fossil-calcifying cyanobacteria *Renalcis* and *Epiphyton* (Table 2, Rowland and Gangloff^104^). Studies on the calcifying phytoplankton *Emiliania huxleyi* suggest that the coccolith layer shields the organism from damaging ultraviolet radiation (UV-R), which could inhibit their calcification and photosynthetic processes.^104^^,^^105^^,^^106^ Popall et al.^107^ concluded that the cyanobacterium *Geitlerinema* sp. exhibits high resilience to UV-C radiation through mediated biomineralization, thereby protecting the underlying microbial community. Another study linked biogenic calcification of the cyanobacterium *Rivularia* sp. with seasonal changes and the corresponding intensity of irradiance and photosynthetic activity.^108^ However, it remains uncertain whether these observations truly reflect an evolutionary benefit of CaCO_3_ sheath production. For example, calcifying cyanobacteria from shallow waters, such as those contributing to stromatolite formation, are exposed to high light, providing support for this hypothesis,^109^ whereas terrestrial calcifying cyanobacteria are predominantly exposed to low light conditions in their natural habitats, such as caves.^22^^,^^23^^,^^26^ In addition, cyanobacteria, which are frequently part of biocrusts of semi-arid and arid drylands, are exposed to often extreme high light conditions. The stress of living in high light environments could induce the secretion of sheath material necessary for precipitation,^110^ and a calcified sheath might physically shade the filament. However, high light intensities might also affect precipitation via an induction of the bicarbonate pump.^111^ Some of the absorbed light energy is utilized for the maintenance of the pump, thus preventing the damage of the photosynthetic apparatus. Calcification would then occur as a secondary consequence of the high radiation. However, this scenario is not very likely since the formation of CaCO_3_ sheaths of biocrust cyanobacteria comparable to those found in e.g., *Scytonema* or *Geitleria* from caves has not been observed so far.
(3) Enhanced photosynthetic efficiency by light trapping (Figure 6 top left): a third theory directly challenges the previous one by suggesting that calcification might enhance photosynthesis.^88^ Two mechanisms support this idea such as (1) an increase in CO_2_ partial pressure and (2) light scattering effects of the mineral sheaths (Figure 6). Studies have shown a net increase in CO_2_ partial pressure at the intracellular site of photosynthetic CO_2_ fixation, resulting from an extracellular reduction of total alkalinity by calcification in coccolithophores.^88^ The acceleration of photosynthesis by calcification as part of the CCM has been frequently suggested in independent studies, with evidence speculated for the coccolithophore *E. huxleyi*.^112^^,^^113^ However, other research has failed to confirm this idea.^114^ Recent findings show that the *E. huxleyi* stops calcification under limited dissolved inorganic carbon (DIC) availability but continues photosynthesis at similar rates, suggesting competition and regulation between CCM and calcification and a differential use of carbon for both processes.^115^ However, applying these findings to cyanobacteria, especially terrestrial ones, may be impractical as cyanobacterial calcification does not occur in coccolith vesicles, and the rerouting of carbon as DIC toward CCM and calcification could differ from coccolithophores. Another idea proposing enhanced photosynthetic efficiency suggests that light scattering from calcified sheaths in coccolithophores could help to funnel photons into the cells^116^ or redirect non-directly absorbed photons to nearby cells by scattering in communities, such as deep-water coral reefs.^117^ A light entrapment mechanism exhibited by CaCO_3_ sheaths offers a potential explanation for the common occurrence of biogenic calcification in low-light niches, such as cave environments. Depending on the crystal structure, photons could be guided directly into the cells, for instance, by orthorhombic aragonite.^25^^,^^118^ This theory could be applicable to terrestrial cyanobacteria, which typically grow in biofilms harboring different cyanobacterial species and could help distribute light sufficiently within the community. In cave environments, for example, where light is naturally low but intense in the far-red region (∼700–750 nm) of the spectrum and less intense in blue wavelengths, aragonite luminescence may help to maintain photon entrapment of longer wavelengths, although this is currently speculative. However, this could be supported by the ability of some cyanobacteria, which produce the pigments chlorophyll_d/f_ to act during far-red light photoacclimation (FaRLiP),^119^ which occurs in cave-dwelling cyanobacteria using the far-red light spectrum.^120^
(4) Detoxification and ion sink (Figure 6 top right): in environments with high Ca^2+^ and HCO_3_^−^ in pore solution, these ions, despite being essential for calcification, can also have harmful effects on the metabolism of cyanobacteria. For example, Ca^2+^ is one of the pharmacologically most active cations, which can suppress growth of certain cyanobacteria.^121^ One possibility might be that the removal of Ca^2+^ from solution by transfer to the solid phase by precipitation forming a highly insoluble deposit may be energetically more economical than pumping it out of the cells into a supersaturated fluid.^89^^,^^90^ Alternatively, it is possible that precipitation might help to buffer the pH rise caused by CO_2_ uptake during photosynthesis, as proposed for characeans and coccolithophores.^90^ This might be due to the OH^−^ ions leading to a rapid increase of pH if they were released into a weakly buffered medium (Figure 7). It is thought that the reaction of OH^−^ with an extracellular HCO_3_^−^ ion going along with CO_3_^2−^ formation and the subsequent precipitation of CaCO_3_ opposes a decrease of pH. Therefore, CaCO_3_ precipitation might allow higher rates of photosynthesis in weakly buffered waters. However, it is worth noting that the occurrence of calcifying cyanobacteria does not seem to be restricted to environments with a low natural buffer capacity for acidification.

It is also possible that CaCO_3_ precipitation could be used as a sink for CO_3_^2−^ ions being inevitably formed as a consequence of OH^−^ release. It is worth noting that carbonate ions cannot be used in photosynthesis. Therefore, it is possible that they may have a negative effect on HCO_3_^−^ uptake (Figure 7) when they are attached to the HCO_3_^−^ transporters and block them.^91^ Therefore, CaCO_3_ precipitation might be a way to protect the pump from the CO_3_^2−^ ions, thus ensuring a constant high rate of photosynthesis by unimpaired DIC uptake comprising the three species CO_2_, HCO_3_^−^, and CO_3_^2^^−^.

We have envisaged calcification in (cyano)bacteria as an adaptive function, but an alternate perspective is also evident. Cosmidis and Benzerara,^121^ for example, revisited this conundrum and speculated whether it is adaptive or not. Calcification clearly impacts the functioning of the microbes, and therefore, it has to be taken into account for, e.g., its ecology. However, biomineralization may be described as an exaptation, which describes a feature that now increases fitness or provides a selective advantage under current conditions, but which had a different function at its origin.^121^^,^^122^

## The cyanobacterial calcification process

### A hard process: Biochemistry of calcification

Calcification, as a multi-parametric process, is highly dependent on environmental conditions such as temperature and humidity that promote nucleation and crystal growth^86^^,^^123^ affecting the depth and uniformity of the carbonate layer formed. Overall, biomineralization of CaCO_3_ by cyanobacteria has traditionally been considered an uncontrolled and extracellular process.^124^ However, this dogma has been challenged by the discovery of several cyanobacterial species forming intracellular amorphous calcium carbonates (ACC).^35^^,^^83^^,^^125^ The simplest explanation for microorganisms serving as "whole-cell biocatalysts" for calcification would be their ability to influence local Ca^2+^ or CO_3_^2−^ saturation states.^126^^,^^127^ All these processes are related to the evolution of a CCM by cyanobacteria as a strategy to overcome the particularly low affinity of the enzyme RubisCO to bind CO_2_.^128^ CCM is defined as the measures that result in higher inorganic carbon (Ci; including CO_2_ and DIC), mostly bicarbonate (HCO_3_^−^) affinity of the intact photosynthetic cell compared to the CO_2_ affinity of its RubisCO. Hence, the CCM works to increase the CO_2_ partial pressure in the vicinity of RubisCO, which allows the enzyme to efficiently perform the carboxylation reaction and at the same time suppresses the oxygenase reaction to a large extent.^91^^,^^129^^,^^130^ Details of the CCM differ between species, but the overall process is driven by a set of CO_2_ and HCO_3_^−^ transporters and the carboxysome, which houses the RubisCO complex (Figure 7). DIC enters the cells mainly via HCO_3_^−^/Na^2+^ symports^131^^,^^132^ but also through diffusion of CO_2_. To limit seepage of CO_2_ from the cell, carbonic anhydrases harboring NADPH dehydrogenase (NDH) complexes in the thylakoid and plasma membranes protonate incoming CO_2_ to HCO_3_ (Figure 7). As a consequence, local pH shifts toward the alkaline range, referred to as the alkalinity engine, have been identified as influential in calcification processes due to the transformation of HCO_3_^−^ into CO_2_, which is fixed by RuBisCO.^4^ Initially observed in microbial mats forming stromatolites, the metabolic activity of cyanobacteria was found to directly affect calcification, crystallographic orientation, and the identity of the polymorph.^4^^,^^133^ Although the crystallographic orientation is mainly influenced by nucleation and growth processes that depend on whether crystals that form at the surface of cells, the identity of the crystal polymorph is mainly controlled by the Mg/Ca ratio (e.g., Müller^134^; Zeyen et al.^135^). Dupraz et al.^4^ notably elucidated the mechanisms of the alkalinity engine, leading to the formation of organominerals such as different CaCO_3_ species, which are formed by or near organisms. The alkalinity engine consists of two parts: (1) an intrinsic component driven by photosynthesis (favoring organomineralization) and respiration (counteracting organomineralization) and (2) an extrinsic component influencing mineralization through processes such as evaporation (increasing compound saturation), degassing (local CO_2_ enrichment), and alkaline water input (pH alteration). To rebalance cytosolic pH, cells activate a Ca^2+^/H^+^ antiport (Figure 7), creating an alkaline microenvironment outside the cell. This metabolically driven pH shift alters the carbonate equilibrium, resulting in increased local extracellular concentrations of HCO_3_^−^ and ultimately CO_3_^2−^.^136^^,^^137^ Thus, cells actively influence the local solubility of both Ca^2+^ by export and CO_3_^2−^ due to the alkaline pH shift, impacting the precipitation of CaCO_3_^4^. In addition to the alkalinity engine, crystal formation can be facilitated through seeded nucleation, where a seed provides a potential site for nucleation at reduced saturation levels.^138^ EPS, secreted by cyanobacteria, are often characterized by negatively charged functional groups on the cell surface that are adept at capturing divalent cations such as Ca^2+^.^139^ It was proposed that providing an organic molecule in a precise array, such as certain negatively charged binding groups on the cell surface or within the EPS, may be crucial for initiating nucleation events.^140^ Here, a passively induced process relying on surface properties of the cyanobacterial cell or their EPS was proposed as nucleation site.^58^^,^^141^ The analysis of calcification abilities in cyanobacterial strains from the genera *Leptolyngbya*, *Scytonema*, *Phormidium*, and *Spirulina* revealed varying types of calcifications among species, indicating that crystal structures and growth are influenced by each organism’s unique physical properties, including structures of negatively charged residues.^137^ However, there are concerns about the choice of microorganisms in this study. It seems unlikely that *Spirulina* species would calcify in their natural habitat of alkaline lakes, as it would result in sinking due to the produced stiff sheaths, leading to loss of mobility.

### The result: Crystal chemistry of calcite and aragonite

The Ca^2+^ and CO_3_^2−^ ions can precipitate as CaCO_3_, often forming different polymorphs, including calcite and aragonite, albeit there are other polymorphs or crystal structures involving both ions. The formation of these two mineral species can be influenced by microbes but also occurs abiotically in soils, sediments, and various rocks.^142^ Aragonite is rarer than calcite and often occurs together with Mg-calcite in coastal regions. Besides microbially influenced, environmental conditions are important in the orientation of the precipitation reaction toward calcite or aragonite (e.g., Zeyen et al.^135^). Pure aragonite, for example, can be found in some lakes when Mg is more abundant than Ca in solutions. In some cases, but not always, this occurs in mineral assemblages resulting from the alteration of Mg-rich rocks such as peridotite or serpentinites.^143^

Aragonite is a mineral that is not commonly found due to its relative instability, as it gradually converts to calcite through a process called aragonite-calcite transformation.^144^ This transformation is observable in marine sediments, where aragonite is lost with increasing depth below the water-sediment interface. The transformation occurs through recrystallization along crystal defects in the presence of water, indicating a dissolution-precipitation mechanism. The growth of larger grains at the expense of smaller ones is driven by minimizing surface energy. Since larger grains have less surface area for a given mass compared to smaller grains, they have a lower surface energy. The reaction is further facilitated by the higher energy and greater solubility of structural compounds at the crystal edges, causing dissolution and redeposition in more favorable locations, which are linked in biocrusts with the water phase. This process leads to the dissolution of small crystals and the accelerated growth of larger crystals. At partly saturated conditions, CaCO_3_ precipitation in water menisci between soil particles can induce cementation and increase aggregate stability.

### Light transmission

**Figure 8:**
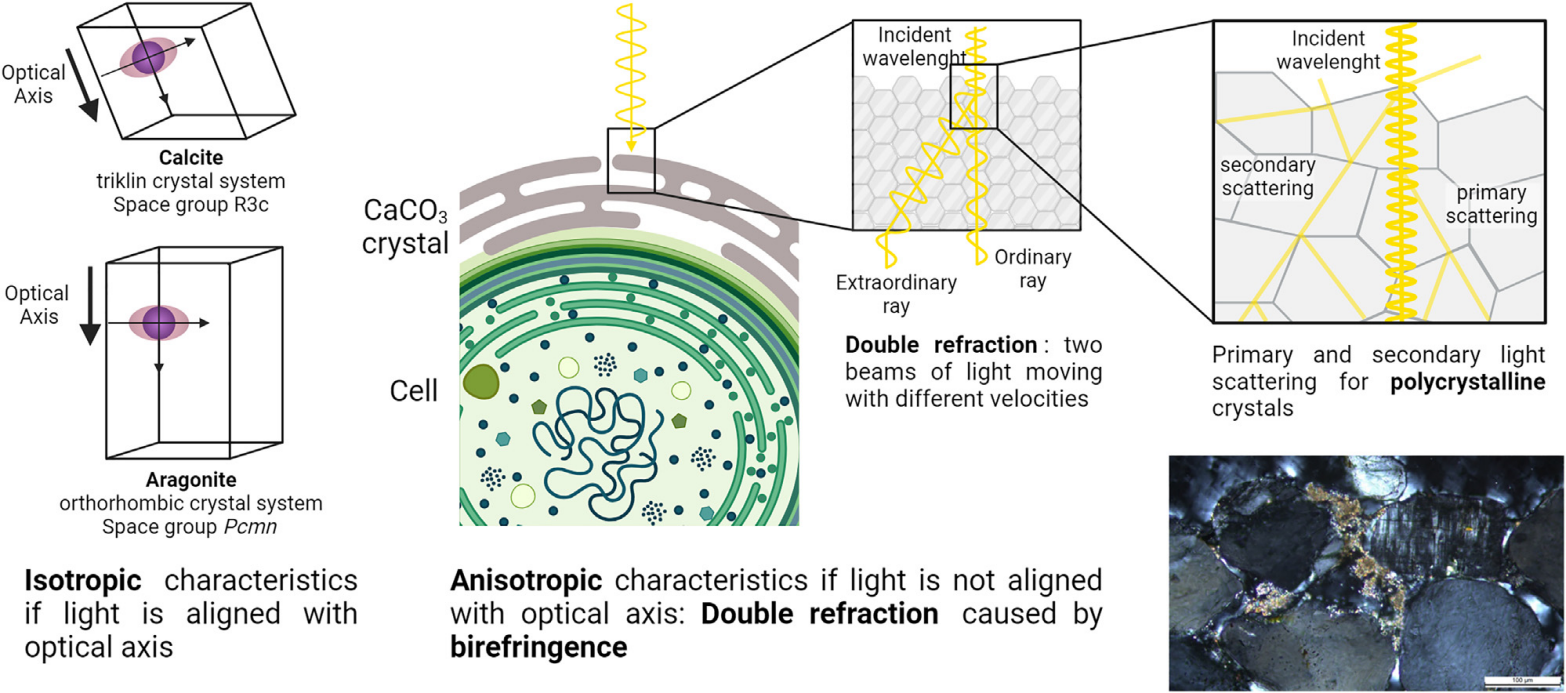
Visualization of the dependency of incident light toward the optical axis of the unit cell of calcite and aragonite, respectively, for the phenomenon of birefringence and the influence of polycrystallinity on light scattering in the crystal sheaths

Calcification in cyanobacteria raises questions regarding the extent to which light transmission is diminished by CaCO_3_ formation around the cell lumen. Minerals like calcite exhibit a crystalline lattice structure that interacts with light based on crystal orientation.^145^ When light enters calcite along the optical axis, it behaves isotropically and travels at a consistent speed (Figure 8). However, off-axis entry results in birefringence, where light splits into two beams with varying speeds due to differing refractive indices depending on direction and polarization. This directional transmission is influenced by the crystal’s anisotropy, symmetry group, and Bravais lattice.

In assessing light propagation in cyanobacteria-formed carbonates, such as in biocrusts, their polycrystalline nature must be acknowledged, with multiple crystals of varying orientations allowing penetration but causing light scattering through diffuse reflection (Figure 8). This scattering occurs internally due to different refractive indices between phases and externally from material surfaces, leading to a decrease in reflected light intensity until dispersion in random directions occurs. Diffuse reflection is also known for organic materials, which arises here from irregularities like cell or fiber boundaries. Organics present at crystal interfaces have typically dimensions comparable to light wavelengths, and photon interaction involves absorption, depending on light wavelength and material properties.

Diminished light propagation at crystallite interfaces with voids can be overcome by saturation with water, which can enhance light transparency by filling voids and reducing internal defects, creating a more homogeneous structure. However, optical transparency with minimal reflection where most light is transmitted is not achieved for polycrystalline CaCO_3_, as it requires an ideal crystal structure.^146^ While single calcite and aragonite crystals offer potential for high optical transmission, polycrystalline forms of CaCO_3_ in sheaths are likely to induce light trapping and hereby decrease photosynthetic activity.

### Surface charging and consequences for adsorption and aggregation

Calcite and aragonite, products of cyanobacterial calcification within biofilms or crusts, involve bacterial cells interacting with stabilizing EPS. Surface charge (SC) at the solid-liquid interface crucially influences particle aggregation and attachment, being responsive to solution parameters like pH, ionic strength, and adsorbing ions.^147^ The point of zero charge (pzc), where SC is neutral, governs particle interactions primarily through van der Waals forces. Organic matter and primary silicates typically exhibit a low pH_pzc_ (<3), contrasting with pure calcite’s pH_pzc_ of around 9.5.^148^ The SC of carbonate minerals is modulated by concentrations of Ca^2+^, Mg^2+^, and CO_3_^2−^, with the isoelectric point hypothesized to be tied to Ca^2+^ and Mg^2+^ concentrations.^149^ Calcite aggregation with negatively charged organic compounds such as those of the EPS at pH < pH_pzc_ is facilitated by attractive opposite charges. Small carbonate particles resulting from cyanobacterial calcification may possess a high specific surface area, offering significant adsorption capacity. The presence of various solution ions can alter surface charge and crystallization potential. Aggregation may be further induced by the sorption of nonpolar organic moieties and hydrophobic interactions, alongside changes in surface roughness and functional groups.^150^^,^^151^^,^^152^ Basically, the surface charge properties of carbonates at the site of formation in calcifying cyanobacteria are still poorly understood, but, based on their potential positive surface charge properties in a predominantly negatively charged matrix of organic compounds, they might have a central role for the stability of the microbial structure.

## Using the process: Modern biotechnological applications of cyanobacterial calcification

### Calcifying cyanobacteria in erosion prevention

It is generally accepted that cyanobacteria excretion of exopolysaccharide in the soil environment supports aggregate formation,^153^ affecting surface hydrology via infiltration or runoff. Depending on abiotic factors arising from the texture of the soil parent material (sand, silt, clay, and unsorted), soil structure, surface roughness, rainfall intensity, and slope gradient, as well as biotic factors such as microbial or specifically cyanobacterial community composition and the presence of biocrusts can either impede or promote runoff. For example, on soils from loess with high shares of silt, infiltration may be reduced due to a greater number of micropores in contrast to soils from sand where infiltration is generally high due to a high share of coarse pores. Specific biotic-abiotic interactions between biocrusts and loess (e.g., exudation of organic polymers, carbonate dissolution and re-precipitation, and dust-dependent metabolic activities of cyanobacteria) that are essential for the formation of stabilized loess have been described.^154^ An exception to this general rule is soils from sand with well-established biocrusts that have high exopolysaccharide content that cause substantial clogging of the surface, thus hindering water infiltration and promoting runoff.^155^ The cyanobacterial-mediated precipitation of calcium carbonate by certain taxa such as *Microcoleus* (Figure 4) could represent a potential factor enhancing the stabilization of intertidal siliciclastic sediments through cementing the sand.^156^ In addition to the effect on erosion caused by exopolysaccharides, biocrusts can impact aggregate stability by formation of CaCO_3_ cements in the pore system. In fact, a large body of literature has emerged in the last decade on MICP that includes cementation of sands to enhance bearing capacity, increase resistance to liquefaction, and improve porous building stones, sequester carbon, stabilization of slopes, and diminishing the risk of soil erosion (e.g., Montoya et al.^157^). However, induction of MICP in desert soils as a mechanism to increase soil stability requires further investigation, standardization, and optimization.^43^ Nevertheless, applications preventing desertification based on biocrust cyanobacteria are on the rise, but they are usually linked to the filamentous structure of the cyanobacteria and the gluing properties of their EPS rather than on calcification processes.^158^ The use of bio-mediated calcium carbonates, for example, has been proposed as a useful bio-protection material for weathered rocks.^159^ Most of these scenarios are inspired by natural biocrusts, which, for example, also stabilize the Great Wall of China against erosion.^160^ A common methodology for using cyanobacteria in erosion prevention is (1) isolating a natural community from, e.g., biocrusts, (2) cyanobacterial upscaling in the laboratory, and (3) the artificial application of the biomass to soil surfaces prone to erosion. Such artificial biocrusts often fail to withstand environmental stress when using this traditional workflow for initial establishment, but recent advances have succeeded by adding, e.g., kaolin^161^ or artificial covers,^162^ which allows positive perspectives.

### Calcifying cyanobacteria in structural engineering

The use of cyanobacterial CaCO_3_ in construction is applicable to completely different areas, such as biocement, LBMs, or alternatives in hardening scenarios. Currently, the largest use of calcium carbonate worldwide is the production of calcium oxide (CaO; approximately 283 million tons annual), commonly known as quicklime or burnt lime, usually by thermal decomposition (calcination or lime burning) of materials such as limestone or seashells containing CaCO_3_ (mineral calcite). The material is produced in a rotary kiln and heated to between 825°C and 1450°C, releasing CO_2_ and leaving behind burnt lime. Although lime is used in small quantities in production processes such as glass, calcium aluminate cement, and organic chemicals, its primary use is in the basic oxygen steelmaking (BOS) process and in the production of cement for the construction industry. Cement production is an energy- and emission-intensive process responsible for approximately 7% of global CO_2_ emissions.^163^ Between 600 kg and 1000 kg of CO_2_ are released per ton of cement.^164^ With regard to the targets set by the Intergovernmental Panel on Climate Change, a distinction is made between material-related and energy-related emissions. Process emissions are primarily the result of heat required to convert CaCO_3_ to CaO + CO_2_. One molecule of CO_2_ is also produced for each molecule of CaO, accounting for approximately 65% of the total emissions.^165^ These raw-material-related emissions are considered fossil emissions due to the release of CO_2_ bound in the stone. Although the emissions from the combustion process can be replaced by renewable sources in the medium term and thus follow the climate pathway, the material-related emissions are considered process-related and therefore unavoidable. These emissions will need to be offset by CO_2_ capture technologies or by replacing fossil limestone with calcium carbonate based on biological processes such as microbial induced carbonate precipitation (MICP) and thus by a biological and climate-friendly process.

For decades there have been approaches using different microorganisms and different metabolic pathways. There are two main types of biocatalysis, a chemoheterotrophic using organic compounds as carbon source and energy supply and an autotrophic pathway using CO_2_ as carbon source and light as energy supply by photosynthesis (photoautotrophic). Heterotrophic pathways include urea hydrolysis (e.g., *Bacillus pasteurii*), denitrification (e.g., *Pseudomonas denitrificans*), ammonification (e.g., Myxobacteria *Myxococcus xanthus*), and methane oxidation (e.g., *Methylocystis parvus*). Autotrophic pathways include photosynthesis by cyanobacteria and MICP by carbonic-anhydrase-producing bacteria (chemoautotrophic) such as *Bacillus megaterium*.^166^

In the case of the heterotrophic pathway, mineralization occurs as a by-product of the metabolic activity of the bacteria. Heterotrophic bacteria utilize organic compounds such as urea, NO_3_^−^, amino acids, or methane for energy and cell material.^167^^,^^168^ An increase in the pH of the surrounding pore water is caused by the metabolic activity of the bacteria, resulting in supersaturated conditions and inducing the precipitation of carbonates in the presence of an inorganic calcium source. The challenge with the heterotrophic pathway is the need for nutrients from other plants, which would inevitably lead to a plate-or-tank competition situation (impact of the growing competition on the use of biomass for energy production) if the process was to be scaled up. CO_2_ is not bound in this biogenic production of CaCO_3_. A life cycle assessment (LCA) shows that process, and raw-material-related production steps lead to CO_2_ emissions, albeit to a lesser extent.^166^ Although it is possible to achieve greenhouse gas neutrality through improved processes, the process cannot be considered a negative emission technology. In addition, the process has a high eutrophication potential, which further limits its scalability.^166^ Phototrophic processes are different since CO_2_ is fixed directly from the air. Highly concentrated CO_2_ sources from industrial processes, such as those used in the production of conventional cement, can be used to increase the growth rate of the microalgae. Cyanobacteria sequester carbon in concrete in the long term, and the most economic route was MICP via cyanobacteria (62 USD kg^−1^ CaCO_3_^166^). To further reduce the material-related emissions from the production of biocement, there are approaches to incorporate the organisms directly as a hardening element without going through the lime production process, although this leads to different material properties, particularly in terms of hardness. Submerged cultivation of cyanobacteria always results in small biomass yields and are often correlated with tremendous energy loss through the necessary mixing and cooling of the biotechnological cultivation systems.^169^ Aerial cultivation, with nebulized medium, reduces the water input by over 80% and increases biomass yields relative to volume.^170^ Some species are naturally exposed to limestone or Ca^2+^-rich sources, grow endolithically, and use bioalkalization causing chemical weathering to access minerals.^66^ All these advantages highlight the importance of investigating cyanobacterial induced calcification in the sector of green and sustainable building materials.

As of today, research has experimented with the incorporation of calcifying microorganisms in the field of sustainable building materials.^171^^,^^172^ Mostly, calcifying ureolytic bacteria, such as *Bacillus* sp. or *Sporosarcina pasteurii*, were used in previous studies for self-healing crack treatment in concrete structures^173^^,^^174^^,^^175^ or used to enhance durability of cement.^172^^,^^176^ These works have demonstrated the feasibility of using microbial-induced carbonate precipitation (MICP) for crack healing purposes. Moreover, applications may extend to soil stabilization or limestone production.^177^ However, ureolytic approaches face the problem of cells having to be provided with urea as substrate for their alkalinity engine, which is not required by autotrophic microorganisms, like cyanobacteria.

**Figure 9:**
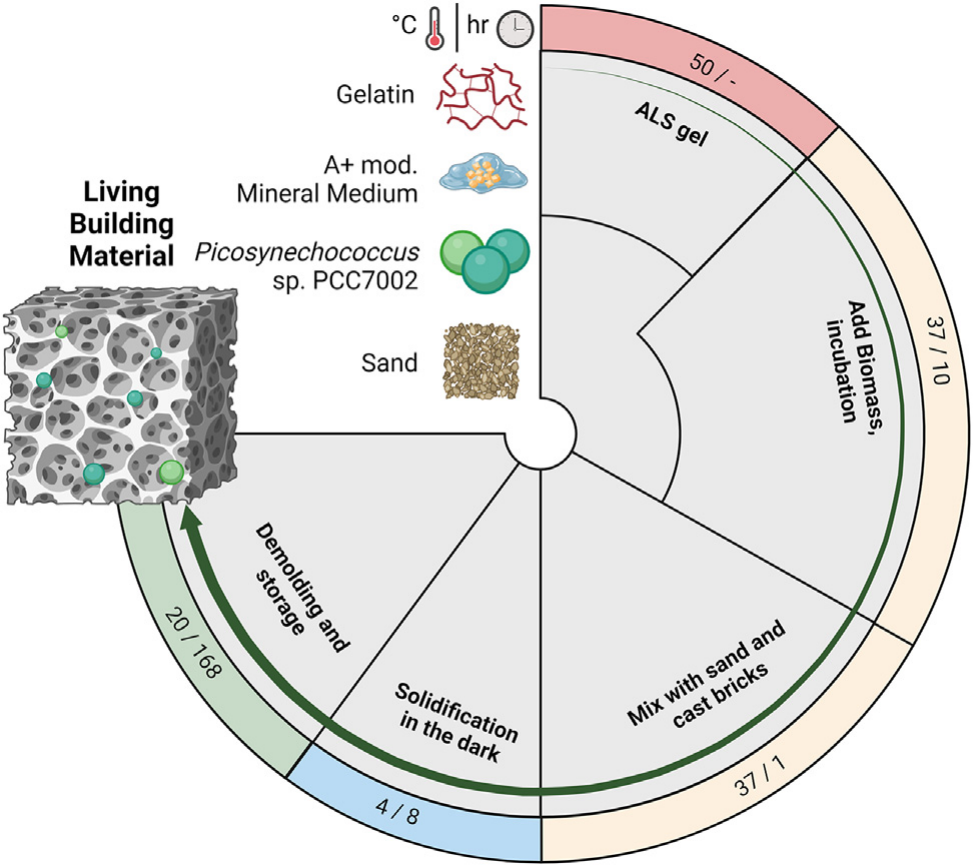
Schematic workflow for the manufacturing of living building materials (LBMs) based on the cyanobacterium model strain *Picosynechococcus* sp. PCC7002 as proposed by Heveran et al.^38^ Briefly, gelatin and a mineral medium are mixed at 50°C (ALS gel), whereas the cyanobacterial biomass is added at 37°C where it is incubated in the light for 10 h. Subsequently, sand is added to the mixture, which can then be casted in molds that solidify during an incubation at 4°C for 8 h in the dark. Afterward, the LBMs can be demolded and stored for a week at ambient conditions until they reach their full stability.

Zhu et al.^178^ initiated the use of autotrophic organisms by discussing potential advantages of using the cyanobacterium *Synechococcus* sp. PCC 8806 for concrete restoration and giving first evidence for the practicability of this idea. Two years later, the authors took their research to an applied level by using carbonate precipitation from *Gloeocapsa* sp. PCC 73107 to achieve higher durability of mortar by microbially induced carbonate precipitation.^179^ Results showed mortar with increased compressive strength but with a decrease in water adsorption and porosity.^179^ Calcifying cyanobacteria such as *Picosynechococcus* sp. PCC 7002 can be used to build actual concrete-like building blocks (Figure 9), which are referred to as LBMs.^37^^,^^38^ They found that “biotically” treated sand samples (sand + gelatine + cyanobacteria + nutrients; Figure 9) showed significantly higher fracture energy compared to abiotically (sand + gelatine + nutrients) treated samples, whereas only small differences in compressive strength were observed. They conclude that the increase in fracture energy is likely a result of a fiber-like effect of the cyanobacteria themselves and that for “the observed overall strengthening, the specific phase of precipitated mineral may be less influential” compared to the effect of gelatin hydrolysis, and therefore the specific toughening mechanisms are still to be identified. The same workflow was recently adjusted using alginate and methylcellulose for extrusion-based bioprinting.^180^

Regarding the vast difference in isolated and characterized cyanobacterial strains able to calcify, using filamentous species could be of benefit for concrete production. With biological fibers added to the concrete for increased tensile strength and enhanced cracking-related properties, filamentous calcifiers could replace these fibers and act as reinforcement within the LBMs.

Another possible application area is in geotechnical engineering. Calcifying cyanobacteria,^38^ ureolytic bacteria,^177^ or isolated enzymes for enzyme-induced carbonate precipitation (EICP)^181^ are able to increase the stiffness and strength and decrease sensitivity to water of raw earth materials.^181^^,^^182^^,^^183^ The most wildly explored method in this context is using microbes with urease activity^177^ or the isolated urease enzyme^183^ and therefore inducing calcite precipitation by urea hydrolysis. This method provides lower costs and a very controlled mechanism although it is currently limited to fine grain soils.^183^

These microbes could be replaced by cyanobacteria, which are more robust with respect to environmental conditions like temperature, salinity, and humidity.^38^ However, it has to be considered that cyanobacteria are often 1–30 μm in diameter,^184^ limiting the penetration into connective pores of soils from fine-sized parent material and high shares of pores <100 nm in diameter. Dilrukshi and Kawasaki^185^ argue that this factor limits the application of cyanobacteria for strengthening soil structure to relatively coarse-grained soil parent materials like sand and gravels. On the other hand, high shares of coarse pores might limit the effect of biomineralization in these coarse-grained materials. One could conclude that different soils might be stabilized using different biological treatments, whether it is EICP or MICP based. In addition, cyanobacteria are known to affect soil structure, which can change porosity,^186^ which might help to create beneficial material properties.

## A possible research roadmap to challenge the one-dimensional dogma

Based on the knowledge gaps identified, the following points need to be considered in future studies on (1) the ecology of terrestrial calcifying cyanobacterial species including an updated experimental design to uncover the benefits of calcified sheaths and (2) further investigations to address the biochemistry of the calcification process to develop a better understanding for (3) applications in terms of increased stability and the production of novel hybrid materials.

(1) Since the vast majority of our current knowledge on cyanobacterial calcification is based on stromatolite-forming, aquatic/marine cyanobacteria with the ability to calcify, the investigation of cave environments is a crucial prerequisite. This will avoid a one-dimensional understanding of the process and ecological significance of the formation of CaCO_3_ sheaths, as is the case in the tubular structures of branching cyanobacteria such as *Scytonema* and *Geitleria,* which have mainly terrestrial habitat niches. Various studies have shown that caves harbor a rich set of cyanobacteria including those that form solidified sheaths,^187^ but they are rarely available in culture collections due to various difficulties.^24^ The specific hunt for such organisms and the identification of possible model strains that will help to understand the calcification process in terrestrial cyanobacteria will allow an update of previous experiments,^72^^,^^188^ leading to more sophisticated insights into the calcification process mediated by terrestrial relatives. Here, the advances for intracellular cyanobacterial Ca^2+^ accumulation should also be considered,^35^ and a combination of experiments will help to uncover the ecological significance of the sheath solidification and therefore tackle the recent theories (Figure 6).
(2) *Biochemistry of the calcification process:* microbial calcification with the formation of CaCO_3_ sheaths around the microbial cells in soils and engineering applications (such as fracture fillings in concrete) does not directly imply an effective cementation of the pore space resulting in an increased water and mechanical stability. Microbial calcification occurs often at some distance to the pore walls, and the contribution of microbial calcification to stability might be weak in the early stages of calcification. In presence of pore water, carbonates are prone to dissolution-precipitation reactions. Precipitation is mainly driven by desiccation and regulated by the partial pressure of CO_2_.^189^ These reactions are temperature dependent. Initially formed CaCO_3_ sheaths can dissolve, and the Ca^2+^ and HCO_3_^−^ ions present in pore solution can be transported by water movement and diffusion. At partial saturation, pore water forms a film on solid surfaces and at the contact point of particles, water menisci form. When the solutes Ca^2+^ and HCO_3_^−^ in the residual water combine again to form CaCO_3_, particle surfaces and particle contacts are cemented and an increase in aggregate stability is likely. In arid climates where rainfall is scarce, carbonates typically accumulate near the soil surface because this is where most evaporation occurs. The intense carbonate precipitation observed at surfaces supported by solution transport and evaporation should also be transferred to building material applications by adjusting water supply using measures of construction. Repeated precipitation of carbonates in connective pore systems narrows and clogs pores and finally can force water to move laterally, which will have a protective effect on building materials. When using carbonates in building materials, a key requirement is that leaching is very limited and only counteracts the loss of carbonates.
  The formation of CaCO_3_ requires the availability of Ca^2+^ ions and the presence of alkaline pH. Here, hydrolysis and protolysis of Ca-containing silicates such as augite, hornblende, epidote, plagioclase, as well as technical by-products such as granulated slag from steel industry and slags from biomass burning have the capability to release Ca^2+^ and consume protons hereby, opposing acidification. For using such silicates in building materials to establish certain Ca^2+^ concentrations and alkaline pH, information on their reaction rate and reactivity in the longer term is needed. Questions arise also regarding the role of Si and Al, which are released during silicate weathering. It is likely that fine-sized secondary mineral phases of Si and Al are formed, which also have a cementing effect too.
  Cyanobacteria and algae colonizing surfaces of building materials accumulate Ca^2+^ from various sources in their cells and EPS-containing biofilms. Following their decay and mineralization, this Ca^2+^ becomes available for CaCO_3_ formation at the surface of building materials. The fact that Ca^2+^ is released here at the surface is interesting as basically high demand for surface stabilization and sealing formation by CaCO_3_ exists here. The availability of minor nutrients such as Zn and Fe and the major nutrient P is assumed to be related to various factors, where carbonates are one potential prominent of them. Here, concentration in the parent material, substrate properties such as pH, organic matter content, the presence of clay minerals, and Fe oxides have to be considered too. Free CaCO_3_ adjusts high pH and hereby reduces the solubility of Zn by enhancing the precipitation of hydroxides.^190^ In calcareous soils, co-precipitation on calcite and chemisorption on Fe oxides are relevant processes explaining Zn fixation.^191^ Moreover, nutrient interactions and antagonisms of Zn, Fe with P exist.^192^ The effect of the immobilization of these elements on biocrust ecology is not well understood. Upon dissolution of carbonates these elements will be released again.
(3) Incorporation of knowledge on the calcification of terrestrial cyanobacteria will help to create a multi-dimensional view on the process allowing for novel applications such as hybrid materials or improve already established applications such as LBMs. The production of LBMs, for example, is currently restricted to the utilization of a single, unicellular marine model strain; however, it is surprisingly not known if it uses calcification in its natural habitat.^37^^,^^38^^,^^180^ This is in addition to certain drawbacks because this strain needs to be grown in a salt-rich medium, for example, which is problematic for most upscaled bioreactor-based approaches due to corrosion.^193^ In addition, the application of filamentous or even branched cyanobacteria for the manufacturing of LBMs might also lead to a more complex matrix and thus to a higher degree of stability. Identifying such strains will also be beneficial for applications aiming for erosion prevention where calcification will add benefits to the concatenation based on EPS alone. The above-described attempts to prevent erosion based on artificial biocrust communities would also be complemented by identifying conditions under which non-calcifying or less-calcifying species such as *Microcoleus*, which are already in use, develop biomineralizing effects. This could be done by manipulating certain culture media (e.g., A+ medium in Heveran et al.^38^) or by the targeted addition of certain substances.^194^

## Conclusion

During the last decades, microbially mediated biomineralization has gained attention in order to understand the ecology of calcifying cyanobacteria, the fundamental biochemical processes, and the subsequent development of applications. However, most of this knowledge has been drawn from aquatic cyanobacteria, which are responsible for the formation of stromatolites, whereas their terrestrial relatives that form tubular structures rather than solidified layers have been neglected. This review not only summarizes the main findings but also adds insights into terrestrial species to provide a multi-dimensional view of the cyanobacterial calcification process. The discussion of often opposing theories regarding the biochemical process or the ecological significance of solidified sheaths in cyanobacteria was intended to stimulate the ongoing debate on these topics, which are of emerging importance due to the growing number of applications. These include erosion control, where artificial biocrusts are used to protect large areas prone to wind erosion. Here, cyanobacteria such as *Microcoleus* already help to stabilize the top centimeter of soils according to biocrust prototypes found in nature. In addition, the manufacturing of LBMs based on the cyanobacterial calcification process as biotechnological platform technology supports the mitigation of CO_2_ emissions in constructional engineering. Also, this methodological workflow can potentially benefit from including concepts drawn from the ecology of terrestrial cyanobacteria capable of forming solidified sheath material. To further drive the latest developments, we have also proposed a multi-faceted research roadmap proposing specific ideas that can help to tackle the identified gaps and further strengthen the development of applications.

## Acknowledgments

The authors want to thank 10.13039/100007569Carl-Zeiss Foundation for funding and the University of Applied Sciences Kaiserslautern for internal funding. We also would like to thank the reviewers for their constructive criticism, which helped to significantly improve the manuscript. N.P. acknowledges financial support from the National Science Foundation: Drylands Critical Zone Network Grant no. EAR-2012475 during the writing and editing of this manuscript. Biocrust thin sections were prepared with the support of White Sands National Park Service Grant no. P21AC11241-01 awarded to N.P. with the help of the Udry lab at 10.13039/100007162UNLV. P.J. also wants to thank Armin Springer for taking the SEM images of *Geitleria* sp. Images were created with Biorender.com.

P.J., L.B.W., C.N., J.F., L.K., J.H., S.D., and G.G. were funded by the 10.13039/100007569Carl-Zeiss Foundation via the project “Cyanobakterien als Baumeister: Maßgeschneiderte Materialien der Zukunft.” P.J. was also funded by the German Research Council (Grit Life JU 3228/1-1). M.L. and L.B.W. were funded by the Ministry of Science and Health, Rhineland-Palatinate (724-0116#2021/004-1501 15405) and the 10.13039/501100002347Federal Ministry of Education and Research (W2V-Strategy2Value, 03WIR4502A). L.B.W. was also funded by an internal grant provided by the University of Applied Sciences Kaiserslautern.

## Author contributions

P.J. designed the study, provided resources and funding, and prepared most figures. All other authors wrote sections and edited the manuscript accordingly.

## Declaration of interests

The authors have no conflict of interest to declare.
